# Determinants of Admission to Critical Care Following Acute Recreational Drug Toxicity: A Euro-DEN Plus Study

**DOI:** 10.3390/jcm12185970

**Published:** 2023-09-14

**Authors:** Roberta Noseda, Matteo Franchi, Alberto Pagnamenta, Laura Müller, Alison M. Dines, Isabelle Giraudon, Fridtjof Heyerdahl, Florian Eyer, Knut Erik Hovda, Matthias E. Liechti, Òscar Miró, Odd Martin Vallersnes, Christopher Yates, Paul I. Dargan, David M. Wood, Alessandro Ceschi, on behalf of the Euro-DEN Plus Research Group

**Affiliations:** 1 Division of Clinical Pharmacology and Toxicology, Institute of Pharmacological Sciences of Southern Switzerland, Ente Ospedaliero Cantonale, 6900 Lugano, Switzerland; roberta.noseda@eoc.ch (R.N.); laura.mueller@eoc.ch (L.M.); 2 Unit of Biostatistics, Epidemiology and Public Health, Department of Statistics and Quantitative Methods, University of Milano-Bicocca, 20126 Milan, Italy; matteo.franchi@unimib.it; 3 National Centre for Healthcare Research and Pharmacoepidemiology, University of Milano-Bicocca, 20126 Milan, Italy; 4 Clinical Trial Unit, Ente Ospedaliero Cantonale, 6900 Lugano, Switzerland; alberto.pagnamenta@eoc.ch; 5 Department of Intensive Care, Ente Ospedaliero Cantonale, 6900 Lugano, Switzerland; 6 Division of Pneumology, University of Geneva, 1211 Geneva, Switzerland; 7 Clinical Toxicology, Guy’s and St Thomas’ NHS Foundation Trust and King’s Health Partners, London SE1 7EH, UK; alison.dines@gstt.nhs.uk (A.M.D.); paul.dargan@gstt.nhs.uk (P.I.D.); david.wood@gstt.nhs.uk (D.M.W.); 8 European Monitoring Centre for Drugs and Drug Addiction (EMCDDA), 1249-289 Lisbon, Portugal; isabelle.giraudon@emcdda.europa.eu; 9 Prehospital Division, Oslo University Hospital, 0424 Oslo, Norway; frihey@gmail.com; 10 The Norwegian Air Ambulance Foundation, 0184 Oslo, Norway; 11 Department of Clinical Toxicology, Klinikum Rechts der Isar, Technical University of Munich, 81675 Munich, Germany; florian.eyer@tum.de; 12 The Norwegian CBRNE Centre of Medicine, Oslo University Hospital, 0450 Oslo, Norway; knuterikhovda@gmail.com; 13 Clinical Pharmacology and Toxicology, University Hospital and University of Basel, 4056 Basel, Switzerland; mliechti@uhbs.ch; 14 Emergency Department, Hospital Clínic, University of Barcelona, 08036 Catalonia, Spain; omiro@clinic.ub.es; 15 Department of General Practice, University of Oslo, 0318 Oslo, Norway; o.m.vallersnes@medisin.uio.no; 16 Oslo Accident and Emergency Outpatient Clinic, City of Oslo Health Agency, 0182 Oslo, Norway; 17 Emergency Department and Clinical Toxicology Unit, Hospital Universitario Son Espases, 07120 Mallorca, Spain; cyatesb@gmail.com; 18 Clinical Toxicology, Faculty of Life Sciences and Medicine, King’s College London, London WC2R 2LS, UK; 19 Faculty of Biomedical Sciences, Università della Svizzera Italiana, 6900 Lugano, Switzerland; 20 Department of Clinical Pharmacology and Toxicology, University Hospital Zurich, 8091 Zurich, Switzerland

**Keywords:** recreational drug use, toxicity, Emergency Department presentation, determinants of critical care admission, Euro-DEN Plus

## Abstract

This study aimed to characterize patients admitted to critical care following Emergency Department (ED) presentation with acute recreational drug toxicity and to identify determinants of admission to critical care. A retrospective multicenter matched case-control study was conducted by the European Drug Emergency Network Plus (Euro-DEN Plus) over the period 2014–2021. The cases were ED presentations with acute recreational drug toxicity admitted to critical care, the controls consisted of ED presentations with acute recreational drug toxicity medically discharged directly from the ED. The potential determinants of admission to critical care were assessed through multivariable conditional stepwise logistic regression analysis and multiple imputation was used to account for the missing data. From 2014 to 2021, 3448 Euro-DEN Plus presentations involved patients admitted to critical care (76.9% males; mean age 33.2 years; SD 10.9 years). Patient age ≥35 years (as compared to ≤18 years) was a determinant of admission to critical care following acute recreational drug toxicity (adjusted odds ratio, aOR, 1.51, 95% confidence interval, CI, 1.15–1.99), along with polydrug use (aOR 1.39, 95% CI 1.22–1.59), ethanol co-ingestion (aOR 1.44, 95% CI 1.26–1.64), and the use of gamma-hydroxybutyrate/gamma-butyrolactone (GHB/GBL, aOR 3.08, 95% CI 2.66–3.57). Conversely, lower odds of admission to critical care were associated with the use of cocaine (aOR 0.85, 95% CI 0.74–0.99), cannabis (aOR 0.44, 95% CI 0.37–0.52), heroin (aOR 0.80, 95% CI 0.69–0.93), and amphetamine (aOR 0.65, 95% CI 0.54–0.78), as was the arrival to the ED during the night (8 p.m.–8 a.m., aOR 0.88, 95% CI 0.79–0.98). These findings, which deserve confirmation and further investigation, could contribute to a more complete understanding of the decision-making process underlying the admission to critical care of patients with acute recreational drug toxicity.

## 1. Introduction

According to the 2022 European Drug Report, approximately 83.4 million or 29% of adults (aged 15–64 years) in the European Union are estimated to have ever consumed one or more recreational drugs or new psychoactive substances (NPS) [1]. When recreational drug/NPS use causes acute toxicity, a presentation to the Emergency Department (ED) may occur and additional health interventions, including admission to critical care, may be required, thus placing an increased burden on healthcare resource utilization [2,3].

The decision-making process to admit a patient to critical care is challenging and influenced by multiple factors that vary from country to country and from institution to institution due to differences, for example, in physical environments, policies, guidelines, relationships among relevant parties, and instruments utilized during decisions [4]. With regard to ED presentations with acute recreational drug toxicity, the decision-making process surrounding the admission to critical care could depend, in addition to the clinical examination with an assessment of drug toxicity severity by standardized scoring tools [5], on other factors specifically related to recreational drug use (e.g., the number and types of drugs used and the time of arrival to the ED).

The demographic and clinical characteristics of patients admitted to critical care because of acute drug toxicity have been described in a few studies, generally at single institutions, over restricted periods, and on relatively small patient numbers (from 55 to 157) [2,3,6]. Noteworthy, none of the previous studies assessed the determinants of admission to critical care of patients presenting to the ED with acute recreational drug toxicity. By contrast, the determinants of admission to critical care have been outlined for self-poisoned patients, who deliberately used drugs to attempt suicide, attending the ED of single university hospitals within a limited timeframe [7,8]. Among these, the ingestion of antihypertensive drugs, the ingestion of antipsychotic drugs, male gender, consciousness impairment, and arrival at the ED less than two hours after ingestion were associated with admission to critical care [7,8]. A cohort study of 9679 patients used the Dutch “National Intensive Care Evaluation” (NICE) quality assessment registry to develop a predictive model of the requirement of admission to critical care, showing that respiratory insufficiency, age >55 years, and Glasgow Coma Score (GCS) <6 were the strongest predictors of admission to critical care for drug intoxicated patients for any purposes [9]. A subsequent cohort study of 517 acutely intoxicated patients admitted at a specialized toxicology medical institution in Munich validated that predictive model [10].

In 2013, the European Monitoring Centre for Drugs and Drug Addiction (EMCDDA) and the DPIP/ISEC Programme of the European Union supported the set-up of the European Drug Emergency Network (Euro-DEN); this, originally composed of 16 sentinel EDs across 10 European countries, enabled the systematic collection of data on the ED presentations related to acute recreational drug toxicity [11]. Since then, the Euro-DEN project has expanded (Euro-DEN Plus), with 31 active sentinel EDs collecting data in 21 countries in 2018 [12]. By the end of 2017, 1434 (6%) of the Euro-DEN Plus ED presentations had admission to critical care as the initial disposition from the ED [12].

By querying the Euro-DEN Plus database, this study aims to characterize the patients admitted to critical care following an ED presentation with acute recreational drug toxicity and assess the determinants of admission to critical care specifically related to recreational drug use.

## 2. Materials and Methods

### 2.1. Euro-DEN Plus

This is a retrospective multicenter matched case-control study from the Euro-DEN Plus database. At sentinel EDs, presentations with symptoms and/or signs consistent with acute recreational drug toxicity (terminology used throughout the manuscript according to the Euro-DEN Plus standard operating procedure), with or without ethanol co-ingestion, are identified from ED/medical records based on one or a combination of the following: patient self-reported use of recreational drugs, information on recreational drug use from witnesses, the opinion of the physician assessing the patient and/or the toxicologist reviewing data entry [12]. Noteworthy, not all Euro-DEN Plus sentinel centers perform toxicological analyses routinely or for the purposes of the network itself, in any case without following a standardized procedure. Recreational drugs are identified as those reported by patients or witnesses and possibly, but not necessarily, confirmed by toxicological analysis. They include established illicit recreational drugs of abuse (e.g., cocaine, heroin, cannabis), NPSs, prescription or over-the-counter drugs used for recreational purposes, plants, fungi or herbal/alternative medicines, and industrial and/or domestic products used for recreational purposes. ED presentations with lone ethanol intoxication, with self-harm or suicidal intention, relating to drug withdrawal or to complications of chronic/previous drug use (without evidence of acute recreational drug toxicity), as well as presentations for which a primary emergency evaluation did not occur because the patient was transferred to other wards, are excluded. Data collected in the Euro-DEN Plus database encompass patient demographics, clinical observations on arrival to the ED, names of the drugs used, predefined clinical features of acute recreational drug toxicity, treatment given, and final disposition from the ED. The latter includes medical discharge from the ED, self-discharge, admission to critical care, admission to other hospital wards, admission to a psychiatric ward, and death. All sentinel EDs contributing to the Euro-DEN Plus database have appropriate local ethics approval for data collection. The reporting of this study follows the Strengthening the Reporting of Observational Studies in Epidemiology (STROBE) reporting guideline for the reporting of case-control studies.

### 2.2. Selection of Cases and Controls

Consecutive ED presentations with acute recreational drug toxicity recorded in the Euro-DEN Plus database from 1 January 2014 to 31 December 2021 (8-year period) were included. During this study period, 40 sentinel EDs distributed in 23 European and neighboring countries contributed to the Euro-DEN Plus network.

Cases were ED presentations with acute recreational drug toxicity that were admitted from the ED to critical care. Controls consisted of ED presentations for acute recreational drug toxicity that were medically discharged directly from the ED. The rationale for the choice of the control group was that of comparing a severe outcome of ED presentations with acute recreational drug toxicity (i.e., admission to critical care) with a minor outcome (i.e., medical discharge from the ED). Controls were matched with cases from the same sentinel ED in order to ensure comparability by geographical area, catchment area, and patient management procedures. A 1:1 individual matching (with simple random sampling without replacement) was applied.

In Norway, the Oslo Accident and Emergency Outpatient Clinic (OAEOC) is a primary care emergency clinic that works as a pre-ED in front of the Oslo University Hospital (OUH) ED and three other Oslo hospitals. The Norwegian health system is two-tiered with a gate-keeping function, and patients cannot present directly to a hospital ED but have to be referred by a primary care doctor or triaged to hospital treatment by the ambulance service. Hence, patients to the OUH ED are either seen at the OAEOC first or brought directly by ambulance. The OAEOC does not have a critical care unit, and patients considered too sick for primary care management are transferred to hospital. A total of 19.3% (427/2218) of the patients presenting at the OAEOC with recreational drug toxicity are transferred. A total of 87.7% (243/277) of patients with recreational drug toxicity brought to the OUH ED are admitted to critical care [13]. Consistently, there were no cases (i.e., ED presentations admitted to critical care) from the OAEOC sentinel center, which, by contrast, was the sentinel center with the highest number of controls (Table 1). Conversely, all Norwegian cases were from the OUH sentinel center that had a very small number of controls. Therefore, for the purpose of this study, the ED presentations from both the OAEOC and the OUH sentinel center were combined and controls for the OUH cases were selected from the medically discharged patients including those from the OAEOC sentinel center.

### 2.3. Covariates

Patient demographics, clinical observations on arrival to the ED, drugs used for recreational purposes, and clinical features of acute recreational drug toxicity were compared between cases and controls.

Patient demographics included patient sex, patient age, the calendar year of ED presentation occurrence, and time of arrival to the ED (during the night, defined from 8 p.m. to 8 a.m., and during the weekend, defined from Friday 5 p.m. to Monday 8 a.m.).

Clinical observations on arrival to the ED were cardiac arrest, tachycardia (>100 beats per minute), respiratory rate (defined as bradypnoea if <12 breaths per minute and tachypnoea if >20 breaths per minute), lactate, systolic blood pressure (defined as hypotension if ≤90 mmHg and hypertension if ≥180 mmHg), diastolic blood pressure, hyperthermia (>39 °C), and GCS (categorized as 15/alert, 9–14/drowsy or 3–8/coma).

Recreational drugs were characterized in terms of number (one or >1) and type (evaluated as the exact term recorded in the database and compared between cases and controls when used in ≥5% of ED presentations from the whole Euro-DEN Plus cohort over the 8-year period 2014–2021). Ethanol co-ingestion was also assessed.

Clinical features of acute recreational drug toxicity encompassed a predefined list in the Euro-DEN Plus database that includes: agitation, anxiety, vomiting, seizures, hallucinations, arrhythmias, psychosis, headache, chest pain, palpitations, and cerebellar features.

The following variables selected based on clinical significance and related to the specific context of recreational drug use were assessed as potential determinants of admission to critical care following ED presentations with acute recreational drug toxicity and included in the multivariable regression models: patient sex (female as reference), patient age (≤18 years; reference/19–34 years, young adults/≥35 years, adults) [1], time of arrival to the ED (including during the night and during the weekend, both yes/no), polydrug use (yes/no), ethanol co-ingestion (yes/no), and use of any of the following drugs reported in ≥5% of the Euro-DEN Plus whole cohort of ED presentations: cocaine, cannabis, heroin, gamma-hydroxybutyrate/gamma-butyrolactone (GHB/GBL), amphetamines, and 3,4-methylenedioxymethamphetamine (MDMA) (all yes/no).

### 2.4. Outcomes

The primary outcome was the association between potential determinants and admission to critical care, measured as odds ratio (OR).

### 2.5. Statistical Analysis

Means and standard deviations (SDs) were used to summarize continuous variables, whereas absolute numbers and percentages were used for categorical variables. Absolute standardized differences were calculated for the comparisons of the covariates of interest between cases and controls. An absolute standardized difference >0.1 was interpreted as a meaningful difference [14]. Multivariable conditional stepwise logistic regression was used to model the association between the aforementioned potential determinants and admission to critical care, with patient sex and age included as fixed covariates. In addition to the complete case analysis, to account for missing data, sensitivity analysis by multiple imputation (under the missing at random assumption) was performed in order to assess the robustness of the results [15]. Adjusted odds ratios (aOR) and 95% confidence intervals (CI) were derived for all potential determinants in all the logistic regression models used. Analyses were performed using the Statistical Analysis System Software (version 9.4; SA Institute, Cary, NC, USA). Differences were considered statistically significant if the 95% CI interval of the aOR did not contain the value 1.

## 3. Results

During the 8-year period between 1 January 2014 and 31 December 2021, there were 61,274 Euro-DEN Plus acute recreational drug toxicity presentations (Table S1). The mean patient age was 33.2 years (SD 10.9 years), with 32,330 (52.8%) of the patients aged between 19 and 34 years. A total of 46,993 (76.7%) presentations involved males. Cocaine, cannabis, heroin, GHB/GBL, amphetamines, and MDMA were the most frequently used drugs (being reported in ≥5% of presentations). Ethanol co-ingestion was recorded in 24,674 (40.3%) presentations. Across the whole cohort, and regardless of the drug(s) used for recreational purposes, agitation was the most frequent clinical feature of acute drug toxicity reported in 16,207 (26.5%) presentations (Table S1).

Out of 61,274 presentations, 3448 (5.6%) had, as the final disposition from the ED, admission to critical care, whereas 37,306 (60.9%) presentations were medically discharged. Table 1 shows the case and (unmatched) control distribution by sentinel center.

### 3.1. Baseline Characteristics

Most of the patients among both the cases and controls were males (2653, 76.9% and 2679, 77.7%, absolute standardized difference 0.02) (Table 2). The cases involved a higher proportion of those aged ≥35 years than the controls (1633, 47.4%, vs. 1369, 39.7%, absolute standardized difference 0.15). At the ED presentation, the cases compared to the controls were more likely to have bradypnoea (<12 breaths per minute, 519, 15.1%, vs. 245, 7.1%, absolute standardized difference 0.28) or tachypnoea (>20 breaths per minute, 590, 17.1%, vs. 262, 7.6%, absolute standardized difference 0.32), and were more frequently in a coma status (GCS 3–8, 1653, 47.9%, vs. 212, 6.2%, absolute standardized difference 1.09). The use of GHB/GBL was more frequently reported among the cases (1143, 33.2%, vs. 409, 11.9%, absolute standardized difference 0.53), whereas cannabis and heroin were more frequently used by the controls (376, 10.9%, vs. 769, 22.3%, absolute standardized difference 0.31 for cannabis, and 585, 17.0%, vs. 744, 21.6%, absolute standardized difference 0.12 for heroin). The differences between the cases and controls in the profile of the clinical features of acute recreational drug toxicity are resumed in Table 2.

### 3.2. Determinants of Admission to Critical Care

**Figure 1:**
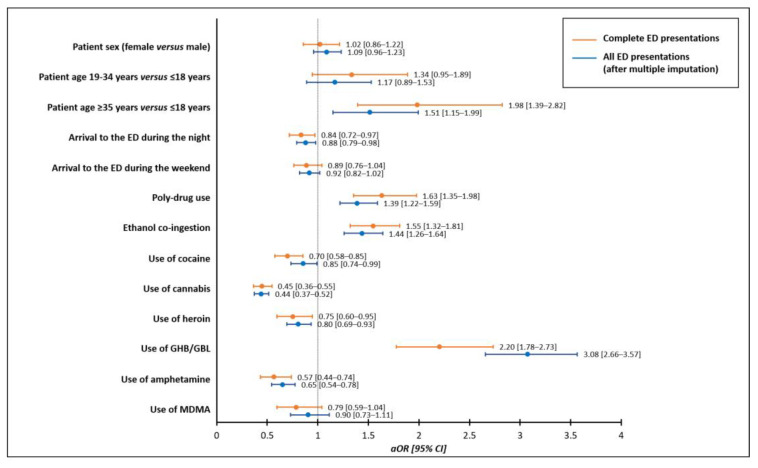
Multivariable conditional stepwise logistic regression analyses on the association of potential determinants of admission to critical care following emergency department presentation due to acute recreational drug toxicity compared to medical discharge from the emergency department. Abbreviations: ED, Emergency Department; GHB/GBL, gamma-hydroxybutyrate/gamma-butyrolactone; MDMA, 3,4-methylenedioxymethamphetamine; aOR, adjusted odds ratio; CI, confidence interval.

In the multivariable conditional stepwise logistic regression analysis on the association of the potential determinants of admission to critical care following acute recreational drug toxicity, patient age ≥35 years, compared to ≤18 years, increased the odds of admission to critical care (aOR 1.98, 95% CI 1.39–2.82) while no effects were observed with regard to patient sex (aOR 1.02, 95% CI 0.86–1.22). The additional determinants of admission to critical care were polydrug use (aOR 1.63, 95% CI 1.35–1.98), ethanol co-ingestion (aOR 1.55, 95% CI 1.32–1.81), and the use of GHB/GBL (aOR 2.20, 95% CI 1.78–2.73). Conversely, lower odds of admission to critical care were associated with the use of cocaine (aOR 0.70, 95% CI 0.58–0.85), cannabis (aOR 0.45, 95% CI 0.36–0.55), heroin (aOR 0.75, 95% CI 0.60–0.95), and amphetamines (aOR 0.57, 95% CI 0.44–0.74), as was the arrival to the ED during the night (8 p.m.–8 a.m., aOR 0.84, 95% CI 0.72–0.97) (Figure 1). The conditional logistic regression model fitted with the same covariates after multiple imputations confirmed these results (Figure 1).

## 4. Discussion

This retrospective matched case-control study from the Euro-DEN Plus identified several determinants of admission to critical care following an ED presentation because of acute recreational drug toxicity. Patient age ≥35 years, polydrug use, ethanol co-ingestion, and the use of GHB/GBL increased the odds of admission to critical care. By contrast, the use of cocaine, cannabis, heroin, amphetamines, and the presentation to the ED during the night were less frequently associated with admission to critical care.

### 4.1. Characteristics of the Whole Euro-DEN Plus Cohort over the 8-Year Period 2014–2021

Recreational drug use is a widespread phenomenon in Europe and given the increasingly frequent use of combinations of multiple drugs [1], it is likely that a number of users will suffer acute drug toxicity possibly followed by the presentation to an ED. The Euro-DEN Plus database collates data on ED presentations with acute recreational drug toxicity from sentinel centers across a wide geographical area in Europe and neighboring countries [11]. Therefore, it represents a data source of paramount importance that can contribute to monitoring and characterizing the European drug situation. During the 8-year period of 2014–2021, over 60 thousand ED presentations were recorded in the Euro-DEN Plus database, mostly involving patients aged 19–34 years, of male sex and most presenting with acute drug toxicity associated with the use of cocaine, cannabis, heroin, GHB/GBL, amphetamines, and MDMA. This is triangulated with data from the 2022 European Drug Report, in which a range of complementary sources demonstrate that these are the most commonly used illicit substances in the last year in Europe [1].

### 4.2. Characteristics of the Euro-DEN Plus Cohort Admitted to Critical Care

In terms of the consequences of the use of drugs for recreational purposes, almost 6% of the Euro-DEN Plus ED presentations required admission to critical care. Previous studies describe the demographic and clinical characteristics of the patients admitted to critical care with acute toxicity caused by drug abuse; however, these were single-center studies over restricted periods and involved small patient groups [2,3,6]. Overall, these studies observed that males were more frequently involved and most of the patients were middle-aged [2,3,6]. The single-center 6-month study conducted by Orsini et al. in a general, community hospital located in Northern Brooklyn, NY, USA, showed that, among 55 patients with clinical signs suggestive of recreational drug use admitted to critical care, the majority used more than one drug. Opioids, cocaine, methadone, benzodiazepines, and cannabis were the most commonly identified substances and the admissions occurred most frequently during the week [6]. In a larger and more geographically diverse cohort, over a longer period, and benefiting from the collection of a systematic dataset, our study has, on the one hand, confirmed some of the characteristics previously observed and, on the other, added new information. We found that the patients admitted to critical care after presenting to the ED because of acute recreational drug toxicity were mostly male, most frequently aged 19 to 34 years, reported polydrug use, and presented to the ED at night and during the week. Moreover, these patients were more commonly drowsy/comatose on ED arrival and presented with a range of significant adverse clinical features, most commonly agitation.

### 4.3. Determinants of Admission to Critical Care

In our study, the broad geographical coverage of the Euro-DEN Plus represents an advantage compared to single-center studies because of the much larger sample size and generalizability of the findings; nevertheless, the assessment of the determinants of admission to critical care compared to a medical discharge directly from the ED demands comparability by the geographical area, catchment area, and patient management procedures. To this aim, we applied matching by sentinel center.

Although male sex was previously identified as a determinant of admission to critical care among self-poisoned patients who attempted suicide [7,8], we did not confirm this finding and observed that patient sex was not a determinant of admission to critical care in presentations to the ED with acute recreational drug toxicity. A recent retrospective single-center study at a specialized clinical toxicology unit showed that male sex increased the odds of a severe course of self-poisoning with suicidal intention [16], confirming the evidence that suicide-related behaviors in males, including self-poisoning, are often characterized by a more serious outcome [17,18,19]. Conversely, with regard to drug use for recreational purposes, a previous study by Miro et al. within the Euro-DEN Plus database showed that when the admission to critical care was considered a marker of severity of acute recreational drug toxicity, no sex differences emerged from regression analyses adjusted by age, regardless of the type and number of drugs used [20]. By contrast, patient age ≥35 years was associated with increased odds for admission to critical care, as was polydrug use and ethanol co-ingestion. Within the Euro-DEN Plus database, the co-use of ethanol in patients with central nervous system depressant intoxication has been shown to increase the risk of adverse effects and was associated with a greater need for medical treatment and critical care [21].

Among the most commonly reported recreational drugs within the Euro-DEN Plus whole cohort, GHB/GBL was the one associated with increased odds of admission to critical care, whereas cocaine, cannabis, heroin, and amphetamines were more frequently associated with being medically discharged from the ED. GHB/GBL are drugs with central nervous system (CNS) depressing effects [22]; as recreational drugs, GHB/GBL provoke euphoric, sexual, stimulant, and relaxant effects [23]. The clinical features of acute GHB/GBL intoxication include potentially life-threatening CNS and respiratory depression [24,25]. Acute GHB/GBL toxicity was most common in those aged 30 to 39 years [26]. We found that the use of GHB/GBL was associated with increased odds of admission to critical care. A previous study in the Euro-DEN Plus database showed that when GHB/GBL was used with ethanol (without additional substances), there was a greater depression of consciousness, a higher need for treatment, a higher rate of admission to critical care, and longer hospital stays [27]. Lastly, arriving at the ED at night (regardless of the day of the week) was associated with reduced odds of admission to critical care. At night, compared with daytime hours, reduced medical services due, among others, to a shortage of personnel and/or less frequent and delayed access to hospital services and resources, could explain this finding. Moreover, for the same reasons, the time of disposition from the ED to critical care of patients with acute recreational drug toxicity might be longer at night than during the day, resulting in critical care admission more likely during daytime hours. Nevertheless, the Euro-DEN Plus database collects information on the total length of hospital stays, including, but without the possibility of differentiating, those in the ED.

### 4.4. Strengths and Limitations

This study has several strengths related to the data source itself. By gathering information from EDs on acute recreational drug toxicity, the Euro-DEN Plus provides a peculiar perspective of the EDs that contributes to monitoring changes in drug consumption patterns and observing the most severe clinical features produced by recreational drugs. Moreover, having a wide geographical coverage allows for a large overview of the current situation of drug use for recreational purposes. The methodological strengths include matching by sentinel center as a guarantee of the findings’ generalizability and multiple imputations to account for the missing data due to voluntary data collection.

This study suffers from some limitations inherent to the nature of the Euro-DEN Plus data source. These include: (i) the nonstandardized procedure used by the sentinel centers to identify ED presentations due to acute recreational drug toxicity, which could result in heterogeneous proportions captured; (ii) the fact that participating countries are represented by one to three sentinel centers primarily located in large cities with different capacities and catchment areas; (iii) the absence of information on the context in which the drug use occurred, on the doses used, on the time gap between the drug use and arrival at the ED, and on the management/treatment(s) received during the stay in the critical care because these data are not collected systematically in the Euro-DEN Plus database; and (iv) the heterogeneity of the reasons for admission of the intoxicated patients to critical care across the sentinel centers. Additional study limitations encompass its retrospective design and the fact that the majority of cases originated from the sentinel center in Oslo, which could have impeded a global European view of the patients admitted to critical care because of acute recreational drug toxicity.

## 5. Conclusions

By providing a broad overview both geographically and temporally, our study corroborates previous findings and adds new information on the determinants of admission to critical care following ED presentation with acute recreational drug toxicity. These include patient age ≥35 years, polydrug use, ethanol co-ingestion, and the use of GHB/GBL. By contrast, the use of cocaine, cannabis, heroin, amphetamines, and the presentation to the ED during the night were more frequently associated with medical discharge from the ED. These findings, which deserve confirmation and further investigation, could contribute to a more complete understanding of the decision-making process surrounding the admission to critical care of patients with acute recreational drug toxicity and could provide useful information to clinicians, policymakers, and other stakeholders on recreational drug use at a high risk of admission to critical care.

## Figures and Tables

**Table 1:** Distribution of the sentinel centers from which cases and controls were selected.

Sentinel Center	Total Number of ED Presentations (2014–2021) n (%), N = 61,274	Cases with Admission to Critical Care n (%), N = 3448	Controls Medically Discharged n (%), N = 37,306
Oslo OAEOC (Norway)	13,508 (22.1)	-	9105 (24.4)
London STH (United Kingdom)	9255 (15.1)	460 (13.3)	4670 (12.5)
Dublin (Ireland)	4305 (7.0)	89 (2.6)	2042 (5.5)
Amsterdam (Netherlands)	3561 (5.8)	99 (2.9)	2893 (7.8)
London KCH (United Kingdom)	3111 (5.1)	115 (3.3)	2169 (5.8)
Antwerp (Belgium)	3007 (4.9)	169 (4.9)	2238 (6.0)
Msida (Malta)	2787 (4.6)	59 (1.7)	738 (2.0)
Mallorca (Spain)	2722 (4.4)	30 (0.9)	2387 (6.4)
Paris (France)	2281 (3.7)	183 (5.3)	1500 (4.0)
Basel (Switzerland)	1940 (3.2)	134 (3.9)	1365 (3.7)
Barcelona (Spain)	1529 (2.5)	49 (1.4)	1314 (3.5)
Oslo OUH (Norway)	1527 (2.5)	1394 (40.4)	15 (0.0)
Munich (Germany)	1055 (1.7)	3 (0.1)	212 (0.6)
Bern (Switzerland)	1055 (1.7)	90 (2.6)	639 (1.7)
Gdansk (Poland)	935 (1.5)	2 (0.1)	427 (1.1)
Lugano (Switzerland)	902 (1.5)	103 (3.0)	512 (1.4)
Geneva (Switzerland)	835 (1.4)	53 (1.5)	630 (1.7)
York (United Kingdom)	680 (1.1)	7 (0.2)	450 (1.2)
Ljubljana (Slovenia)	698 (1.1)	119 (3.5)	351 (0.9)
Vilnius (Lithuania)	686 (1.1)	51 (1.5)	453 (1.2)
London SMH (United Kingdom)	675 (1.1)	10 (0.3)	517 (1.4)
Ghent (Belgium)	636 (1.0)	35 (1.0)	343 (0.9)
Tallin (Estonia)	619 (1.0)	43 (1.3)	488 (1.3)
Bratislava (Slovakia)	408 (0.7)	23 (0.7)	196 (0.5)
Sofia (Bulgaria)	363 (0.6)	-	357 (1.0)
Drogheda (Ireland)	322 (0.5)	23 (0.7)	96 (0.3)
Riga (Latvia)	272 (0.4)	-	264 (0.7)
Helsinki (Finland)	253 (0.4)	-	203 (0.5)
Monza (Italy)	208 (0.3)	2 (0.1)	147 (0.4)
Copenhagen (Denmark)	155 (0.3)	14 (0.4)	56 (0.2)
Parnu (Estonia)	171 (0.3)	20 (0.6)	104 (0.3)
Kaunus (Lithuania)	164 (0.3)	1 (0.0)	100 (0.3)
Roskilde (Denmark)	155 (0.3)	18 (0.5)	94 (0.3)
Utrecht (Netherlands)	135 (0.2)	39 (1.1)	71 (0.2)
Bucharest (Romania)	133 (0.2)	-	18 (0.1)
Prague (Czech Republic)	70 (0.1)	8 (0.2)	17 (0.1)
Tbilisi (Georgia)	68 (0.1)	-	68 (0.2)
Izmir (Turkey)	54 (0.1)	2 (0.1)	30 (0.1)
Rozzano (Italy)	26 (0.0)	1 (0.0)	20 (0.1)
Nicosia (Italy)	8 (0.0)	-	7 (0.0)

Abbreviations: ED, Emergency Department; Oslo OAEOC, Oslo Accident and Emergency Outpatient Clinic; London STH, St Thomas’ Hospital; London KCH, King’s College Hospital; Oslo OUH, Oslo University Hospital; London SMH, London St Mary’s Hospital.

**Table 2:** Comparison of baseline characteristics between emergency department presentations admitted to critical care (cases) and presentations medically discharged directly from the emergency department (controls) gathered in the Euro-DEN Plus database over the 8-year period 2014–2021.

	Cases n (%), N = 3448	Matched Controls n (%), N = 3448	Absolute Standardized Difference
Patient demographics			
Patient sex			
Male	2653 (76.9)	2679 (77.7)	0.02
Female	795 (23.1)	768 (22.3)	
Missing	-	1 (0.0)	
Patient age, years			
Mean (SD)	35.1 (10.9)	33.0 (11.0)	0.19
≤18	112 (3.3)	191 (5.5)	0.11
19–34	1685 (48.9)	1865 (54.1)	0.11
≥35	1633 (47.4)	1369 (39.7)	0.15
Missing	18 (0.5)	23 (0.7)	
Calendar year of ED presentation occurrence			
2014	316 (9.2)	275 (8.0)	0.04
2015	308 (8.9)	325 (9.4)	0.02
2016	352 (10.2)	271 (7.9)	0.08
2017	458 (13.3)	399 (11.6)	0.05
2018	607 (17.6)	532 (15.4)	0.06
2019	527 (15.3)	549 (15.9)	0.02
2020	500 (14.5)	594 (17.2)	0.08
2021	380 (11.0)	503 (14.6)	0.11
Time of arrival at the ED			
During the night (from 8 p.m. to 8 a.m.)	1870 (54.2)	1994 (57.8)	0.07
Missing	9 (0.3)	4 (0.1)	
During the weekend (from Friday 5 p.m. to Monday 8 a.m.)	1555 (45.1)	1622 (47.0)	0.04
Missing	9 (0.3)	4 (0.1)	
Clinical observations on arrival at the ED			
Cardiac arrest	142 (4.1)	3 (0.1)	0.28
Missing	1 (0.0)	7 (0.2)	
Tachycardia (>100 beats per minute)	986 (28.6)	960 (27.8)	0.01
Missing	165 (4.8)	199 (5.8)	
Respiratory rate, breaths per minute			
Bradypnoea (<12)	519 (15.1)	245 (7.1)	0.28
12–20	1695 (49.2)	2192 (63.6)	0.47
Tachypnoea (>20)	590 (17.1)	262 (7.6)	0.32
Missing	644 (18.7)	149 (21.7)	
Lactate, mmol			
<1.3	846 (24.5)	210 (6.1)	0.14
1.3–2	540 (15.7)	262 (7.6)	0.28
>2	1130 (32.8)	300 (8.7)	0.12
Missing	932 (27.0)	2676 (77.6)	
Systolic blood pressure, mmHg			
Mean (SD)	123.9 (25.8)	127.2 (19.9)	0.14
Hypotension (≤90)	245 (7.1)	66 (1.9)	0.24
Hypertension (≥180)	77 (2.2)	41 (1.2)	0.07
91–179	2918 (84.6)	2660 (77.2)	0.24
Missing	208 (6.0)	681 (19.8)	
Diastolic blood pressure, mmHg			
Mean (SD)	76.3 (19.2)	77.1 (14.8)	0.05
Missing	214 (6.2)	684 (19.8)	
Hyperthermia (≥39 °C)	107 (3.10)	2 (0.06)	0.27
Missing	641 (18.59)	654 (18.97)	
Glasgow Coma Score			
Alert	718 (20.8)	1915 (55.5)	0.78
Drowsy	997 (28.9)	1248 (36.2)	0.16
Coma	1653 (47.9)	212 (6.2)	1.09
Missing	80 (2.3)	73 (2.1)	
Drugs used for recreational purposes			
Number of drugs used			
One	2109 (61.2)	2206 (64.0)	0.06
>1	1339 (38.8)	1242 (36.0)	
Mean (SD)	2.5 (0.8)	2.4 (0.7)	0.13
Drugs used in ≥ 5% of the ED presentations from the whole Euro-DEN Plus cohort over the 8-year period 2014–2021			
Cocaine	629 (18.2)	658 (19.1)	0.02
Cannabis	376 (10.9)	769 (22.3)	0.31
Heroin	585 (17.0)	744 (21.6)	0.12
GHB/GBL	1143 (33.2)	409 (11.9)	0.53
Amphetamine	353 (10.2)	458 (13.3)	0.10
MDMA	244 (7.1)	271 (7.9)	0.03
Ethanol co-ingestion	1496 (43.4)	1391 (40.3)	0.23
Missing	1195 (34.7)	926 (26.9)	
Clinical features of acute recreational drug toxicity			
Agitation	1298 (37.7)	767 (22.2)	0.34
Missing	28 (0.8)	24 (0.7)	
Anxiety	1152 (33.4)	586 (17.0)	0.39
Missing	29 (0.8)	28 (0.8)	
Vomiting	479 (13.9)	336 (9.7)	0.13
Missing	28 (0.8)	27 (0.8)	
Seizures	424 (12.3)	74 (2.2)	0.40
Missing	27 (0.8)	28 (0.8)	
Hallucinations	401 (11.6)	191 (5.5)	0.22
Missing	27 (0.8)	28 (0.8)	
Arrhythmias	386 (11.2)	30 (0.9)	0.45
Missing	126 (3.7)	124 (3.6)	
Psychosis	234 (6.8)	131 (3.8)	0.13
Missing	28 (0.8)	26 (0.8)	
Headache	187 (5.4)	142 (4.1)	0.06
Missing	30 (0.9)	27 (0.8)	
Chest pain	175 (5.1)	227 (6.6)	0.07
Missing	29 (0.8)	28 (0.8)	
Palpitations	129 (3.7)	301 (8.7)	0.21
Missing	26 (0.8)	27 (0.8)	
Cerebellar features	79 (2.3)	57 (1.7)	0.04
Missing	1430 (41.5)	1667 (48.4)	

An absolute standardized difference > 0.1 was interpreted as a meaningful difference. Abbreviations: SD, standard deviation; ED, emergency department; GHB/GBL, gamma-hydroxybutyrate/gamma-butyrolactone; MDMA, 3,4-methylenedioxymethamphetamine.

## Data Availability

The dataset used and analyzed during this current study is available from the corresponding author on reasonable request.

## Supplementary Materials

The following supporting information can be downloaded at https://www.mdpi.com/article/10.3390/jcm12185970/s1, Table S1: Characteristics of the whole Euro-DEN Plus cohort over the 8-year period 2014–2021; Table S2: Euro-DEN Plus Research Group Corporate Authors.

## References

[1] (2022). European Monitoring Centre for Drugs and Drug Addiction. European Drug Report. Trend and Developments. http://www.emcdda.europa.eu/.

[2] Westerhausen D., Perkins A.J., Conley J., Khan B.A., Farber M. (2020). Burden of Substance Abuse-Related Admissions to the Medical ICU. Chest.

[3] Cervellione K.L., Shah A., Patel M.C., Curiel Duran L., Ullah T., Thurm C. (2019). Alcohol and Drug Abuse Resource Utilization in the ICU. Subst. Abuse.

[4] Batten J.N., Caruso P., Metaxa V. (2023). More than patient benefit: Taking a broader view of ICU admission decisions. Intensive Care Med..

[5] Zimmerman J.E., Kramer A.A., McNair D.S., Malila F. (2006). Acute Physiology and Chronic Health Evaluation (APACHE) IV: Hospital mortality assessment for today’s critically ill patients. Crit. Care Med..

[6] Orsini J., Din N., Elahi E., Gomez A., Rajayer S., Malik R., Jean E. (2017). Clinical and epidemiological characteristics of patients with acute drug intoxication admitted to ICU. J. Community Hosp. Intern. Med. Perspect..

[7] Beaune S., Juvin P., Beauchet A., Casalino E., Megarbane B. (2016). Deliberate drug poisonings admitted to an emergency department in Paris area—A descriptive study and assessment of risk factors for intensive care admission. Eur. Rev. Med. Pharmacol. Sci..

[8] Novack V., Jotkowitz A., Delgado J., Novack L., Elbaz G., Shleyfer E., Barski L., Porath L. (2006). General characteristics of hospitalized patients after deliberate self-poisoning and risk factors for intensive care admission. Eur. J. Intern. Med..

[9] Brandenburg R., Brinkman S., de Keizer N.F., Kesecioglu J., Meulenbelt J., de Lange D.W. (2017). The need for ICU admission in intoxicated patients: A prediction model. Clin. Toxicol..

[10] Böll R., Romanek K., Schmoll S., Stich R., Ott A., Stenzel J., Geith S., Eyer F., Rabe C. (2018). Independent validation of the ICU requirement score in a cohort of acutely poisoned adults. Clin. Toxicol..

[11] https://www.emcdda.europa.eu/publications/data-factsheet/european-drug-emergencies-network-euro-den-plus-data-and-analysis_en.

[12] European Monitoring Centre for Drugs and Drug Addiction (2020). Drug-Related Hospital Emergency Presentations in Europe: Update from the Euro-DEN Plus Expert Network.

[13] Syse V.L., Brekke M., Grimsrud M.M., Persett P.S., Heyerdahl F., Hovda K.E., Vallersnes O.M. (2019). Gender differences in acute recreational drug toxicity: A case series from Oslo, Norway. BMC Emerg. Med..

[14] Austin P.C. (2009). Using the Standardized Difference to Compare the Prevalence of a Binary Variable between Two Groups in Observational Research. Commun. Stat. Simul. Comput..

[15] Sterne J.A., White I.R., Carlin J.B., Spratt M., Royston P., Kenward M.G., Wood A.M., Carpenter J.R. (2009). Multiple imputation for missing data in epidemiological and clinical research: Potential and pitfalls. BMJ.

[16] Geith S., Lumpe M., Schurr J., Rabe C., Ott A., Zellner T., Rentrop M., Eyer F. (2022). Characteristics and predictive factors of severe or fatal suicide outcome in patients hospitalized due to deliberate self-poisoning. PLoS ONE.

[17] Mergl R., Koburger N., Heinrichs K., Székely A., Tóth M.D., Coyne J., Quintão S., Arensman E., Coffey C., Maxwell M. (2015). What Are Reasons for the Large Gender Differences in the Lethality of Suicidal Acts? An Epidemiological Analysis in Four European Countries. PLoS ONE.

[18] Freeman A., Mergl R., Kohls E., Székely A., Gusmao R., Arensman E., Koburger N., Hegerl U., Rummel-Kluge C. (2017). A cross-national study on gender differences in suicide intent. BMC Psychiatry.

[19] Cibis A., Mergl R., Bramesfeld A., Althaus D., Niklewski G., Schmidtke A., Hegerl U. (2012). Preference of lethal methods is not the only cause for higher suicide rates in males. J. Affect. Disord..

[20] Miró Ò., Burillo-Putze G., Schmid Y., Salgado E., Liechti M., Dines A.M., Giraudon I., Heyerdahl F., Hovda K., Vallersne O.M. (2022). Severity of emergency department presentations due to acute drug toxicity in Europe: A longitudinal analysis over a 6-year period (2014–2019) stratified by sex. Eur. J. Emerg. Med..

[21] Heier E.C., Eyer F., Rabe C., Geith S., Dargan P.I., Wood D.M., Heyerdahl F., Dines A.M., Giraudon I., Erik Hovda K. (2022). Clinical effect of ethanol co-use in patients with acute drug toxicity involving the use of central nervous system depressant recreational drugs. Eur. J. Emerg. Med..

[22] Baselt R.C. (2017). Disposition of Toxic Drugs and Chemicals in Man.

[23] Tay E., Lo W.K.W., Murnion B. (2022). Current Insights on the Impact of Gamma-Hydroxybutyrate (GHB) Abuse. Subst. Abuse Rehabil..

[24] Schep L.J., Knudsen K., Slaughter R.J., Vale J.A., Mégarbane B. (2012). The clinical toxicology of γ-hydroxybutyrate, γ-butyrolactone and 1,4-butanediol. Clin. Toxicol..

[25] Darke S., Peacock A., Duflou J., Farrell M., Lappin J. (2020). Characteristics and circumstances of death related to gamma hydroxybutyrate (GHB). Clin. Toxicol..

[26] Miró Ò., Waring W.S., Dargan P.I., Wood D.M., Dines A.M., Yates C., Giraudon I., Moughty A., O’Connor N., Heyerdahl F. (2021). Variation of drugs involved in acute drug toxicity presentations based on age and sex: An epidemiological approach based on European emergency departments. Clin. Toxicol..

[27] Galicia M., Dargan P.I., Dines A.M., Yates C., Heyerdahl F., Hovda K.E., Giraudon I., Wood D.M., Miró Ò., Euro-DEN Plus Research Group (2019). Clinical relevance of ethanol coingestion in patients with GHB/GBL intoxication. Toxicol. Lett..

