# Supplementary material for: Determinants of Admission to Critical Care Following Acute Recreational Drug Toxicity: A Euro-DEN Plus Study
Source: J Clin Med. 2023 Sep 14;12(18):5970. doi: 10.3390/jcm12185970 (PMC10532086; doi:10.3390/jcm12185970)
Supplement: Supplementary file 1 [file jcm-12-05970-s001.zip › Table S1.pdf]

Table S1 Characteristics of the whole Euro-DEN Plus cohort over the 8-year period 2014-2021.

	**n(%), N=61274**
Patient sex	
Male	46993 (76.7)
Female	14274 (23.3)
Missing	7 (0.0)
Patient age, years	
Mean [SD]	33.2 [10.9]
≤18	3534 (5.8)
19-34	32330 (52.8)
≥35	24812 (40.2)
Missing	598 (1.0)
Calendar year of ED presentation occurrence	
2014	5444 (8.9)
2015	5265 (8.6)
2016	5324 (8.7)
2017	7914 (12.9)
2018	9901 (16.2)
2019	9785 (16.0)
2020	9343 (15.3)
2021	8298 (13.5)
Drugs reported in ≥5% of ED presentations	
Cocaine	13359 (21.8)
Cannabis	13236 (21.6)
Heroin	10987 (17.9)
GHB/GBL	7670 (12.5)
Amphetamine	6021 (9.8)
MDMA	4772 (7.8)
Unknown	3406 (5.6)
Ethanol co-ingestion	
Yes	24674 (40.3)
No	21226 (34.6)
Missing	15374 (25.1)
Clinical features of acute recreational drug toxicity	
Agitation	
Yes	16207 (26. 5)
No	44006 (71.8)
Missing	1061 (1.7)

Anxiety	
Yes	12358 (20.2)
No	47858 (78.1)
Missing	1058 (1.7)
Vomiting	
Yes	5786 (9.4)
No	54392 (88.8)
Missing	1096 (1.8)
Seizures	
Yes	2338 (3.8)
No	57852 (94.4)
Missing	1084 (1.8)
Hallucinations	
Yes	4604 (7.5)
No	55597 (90.7)
Missing	1073 (1.8)
Arrhythmias	
Yes	1151 (1.9)
No	56766 (92.6)
Missing	3357 (5.5)
Psychosis	
Yes	4551 (7.4)
No	55646 (90.8)
Missing	1077 (1.8)
Headache	
Yes	2667 (4.4)
No	57537 (93.9)
Missing	1070 (1.8)
Chest pain	
Yes	5181 (8.5)
No	55006 (89.8)
Missing	1087 (1.8)
Cerebellar features	
Yes	1082 (1.8)
No	31712 (51.8)
Missing	28480 (46.5)
Palpitations	
Yes	5280 (8.6)
No	54918 (89.6)

Missing	1076 (1.8)
Final disposition from the ED	
Medical discharge	37306 (60.9)
Self-discharge	8653 (14.1)
Admission to critical care	3448 (5.6)
Admission to other hospital wards	8122 (13.3)
Admission to a psychiatric ward	3242 (5.3)
Death	84 (0.1)
Unknown	419 (0.7)

Abbreviations: ED, emergency department; SD, standard deviation; GHB/GBL, gamma-hydroxybutyrate/gamma-butyrolactone; MDMA, 3,4-methylenedioxymethamphetamine
