# Supplementary material for: Determinants of Admission to Critical Care Following Acute Recreational Drug Toxicity: A Euro-DEN Plus Study
Source: J Clin Med. 2023 Sep 14;12(18):5970. doi: 10.3390/jcm12185970 (PMC10532086; doi:10.3390/jcm12185970)
Supplement: Supplementary file 1 [file jcm-12-05970-s001.zip › Table S2.pdf]

Table S2: Euro-DEN Plus Research Group Corporate Authors:

Monia Aloise, Jacek Anand, Lukasz Anand, Kurt Anseeuw, Erato Antoniou, Robertas Badaras, George Bailey, Jeffrey Bonnici, Miran Brvar, Carl Byrne, Blazena Caganova, Feriyde Calýskan, Laurence Daveloose, Miguel Galicia, Birgit Gartner, Johan Gillebeert, Ketevan Gorozia, Damjan Grenc, Femke Gresnigt, Laura Hondebrink, Gesche Jürgens, Jutta Konstari, Jan Kruczynski, Soso Kutubidze, Gabija Laubner, Evangelia Liakoni, Viesturs Liguts, Cathelijne Lyphout, Bruno Mégarbane, Adrian Moughty, Aymen M'Rad, Gabriela Viorela Nitescu, Niall O'Connor, Raido Paasma, Juan Ortega Perez, Marius Perminas, Per Sverre Persett, Kristiina Põld, Erik Puchon, Jordi Puiguriguer, Julia Radenkova-Saeva, Jan Rulisek, Caroline Samer, Maro H Sandel, Yasmin Schmid, Irene Scholz, Roberts Stašinskis, Jonas Surkus, Irma van den Hengel-Koot, Federico Vigorita, Severin B Vogt, William Stephen Waring, Ioanna Yiasemi, Sergej Zacharov, Tobias Zellner

First name	Middle name	Last/family name	Affiliation
Monia		Aloise	Emergency Department, Humanitas Research Hospital, via Manzoni 56, 20089 Rozzano, Italy
Jacek	Sein	Anand	1. Pomeranian Centre of Toxicology, Gdansk, Poland<br>2. Medical University Gdansk, Department of Clinical Toxicology, Gdansk, Poland
Lukasz	Sein	Anand	Pomeranian Centre of Toxicology, Gdansk, Poland
Kurt		Anseeuw	Department of Emergency Medicine, ZNA Stuivenberg, Antwerp, Belgium
Erato		Antoniou	Emergency Department, Nicosia General Hospital, Nicosia, Cyprus
Robertas		Badaras	Republic Vilnius University Hospital, Vilnius, Lithuania
George		Bailey	Emergency Department, St Mary's Hospital, Imperial College Healthcare NHS Trust, London, UK
Jeffrey		Bonnici	Mater Dei Hospital, Msida, MSD 2090, Malta
Miran		Brvar	Centre for Clinical Toxicology and Pharmacology, University Medical Centre Ljubljana, Ljubljana, Slovenia
Carl		Byrne	Department of Emergency Medicine, Our Lady of Lourdes Hospital, Drogheda, County Louth, Republic of Ireland
Blazena		Caganova	National Toxicological Information Centre, University Hospital, Bratislava, Slovakia
Feriyde		Calýskan	Izmir Medical Sciences University, Tepecik Training and Research Hospital, Izmir, Turkey
Laurence		Daveloose	Emergency Department, Ghent University Hospital, Belgium
Miguel		Galicia	Emergency Department, Hospital Clinic Barcelona Spain
Birgit		Gartner	Emergency Department, Geneva University Hospitals, Rue Gabrielle-Perret-Gentil 4, Switzerland
Johan		Gillebeert	Department of Emergency Medicine, ZNA Stuivenberg, Antwerp, Belgium
Ketevan		Gorozia	Archangel St. Michael Multiprofile Clinical Hospital, Tbilisi, Georgia
Damjan		Grenc	Centre for Clinical Toxicology and Pharmacology, University Medical Centre Ljubljana, Ljubljana, Slovenia
Femke	MJ	Gresnigt	Emergency department, OLVG Hospital, Amsterdam, The Netherlands
Laura		Hondebrink	Dutch Poisons Information Center, University Medical Center, Utrecht University, Utrecht, The Netherlands
Gesche		Jürgens	Zealand University Hospital Roskilde, Clinical Pharmacology Unit, Roskilde, Denmark
Jutta		Konstari	Malmi Hospital, Helsinki, Finland
Jan		Kruczynski	Emergency Department, St Mary's Hospital, Imperial College Healthcare NHS Trust, London, UK

Soso		Kutubidze	Archangel St. Michael Multiprofile Clinical Hospital, Tbilisi, Georgia
Gabija		Laubner	Republic Vilnius University Hospital, Vilnius, Lithuania
Evangelia		Liakoni	Clinical Pharmacology and Toxicology, Department of General Internal Medicine, Inselspital, Bern University Hospital, University of Bern, Bern, Switzerland
Viesturs		Liguts	Riga East Clinical University Hospital, Riga, Latvia
Cathelijne		Lyphout	Emergency Department, Ghent University Hospital, Belgium
Bruno		Mégarbane	Department of Medical and Toxicological Critical Care, Lariboisière Hospital, INSERM UMRS-1144, Université Paris Cité, Paris, France
Adrian		Moughty	Emergency Department, Mater Misericordiae University Hospital, Dublin 7, Republic of Ireland
Aymen		M’Rad	Department of Medical and Toxicological Critical Care, Lariboisière Hospital, INSERM UMRS-1144, Paris University, Paris, France
Gabriela	Viorela	Nitescu	Children’s Hospital Grigore Alexandrescu, Bucharest, Romania
Niall		O'Connor	Department of Emergency Medicine, Our Lady of Lourdes Hospital, Drogheda, County Louth, Republic of Ireland
Raido		Paasma	Foundation Pärnu Hospital, Pärnu Estonia
Juan		Ortega Perez	Clinical Toxicology Unit, Emergency Department, Hospital Son Espases, Palma de Mallorca, Balearic Island, Spain
Marius		Perminas	Lithuanian University of Health Sciences, Kaunas, Lithuania
Per Sverre		Persett	Department of Acute Medicine, Medical Division, Oslo University Hospital, Norway
Kristiina		Põld	Emergeny Medicine Department, North-Estonia Medical Centre, Tallinn, Estonia
Erik		Puchon	National Toxicological Information Centre, University Hospital, Bratislava, Slovakia
Jordi		Puiguriguer	Clinical Toxicology Unit, Emergency Department, Hospital Son Espases, Palma de Mallorca, Balearic Island, Spain
Julia		Radenkova-Saeva	University Hospital for Emergency Medicine "N.I.Pirogov”, Sofia, Bulgaria
Jan		Rulisek	Department of Anesthesia and Intensive Care, 1st Faculty of Medicine, Charles University and General University Hospital, U Nemocnice 2, 120 00 Prague 2, Czech Republic
Caroline		Samer	Clinical Pharmacology and Toxicology Department, Geneva University Hospitals, Rue Gabrielle-Perret-Gentil 4, Switzerland
Maro	H	Sandel	Emergency department, OLVG hospital, Amsterdam, The Netherlands
Yasmin		Schmid	Clinical Pharmacology and Toxicology, University Hospital and University of Basel, Basel, Switzerland
Irene		Scholz	Clinical Pharmacology and Toxicology, Department of General Internal Medicine, Inselspital, Bern University Hospital, University of Bern, Bern, Switzerland
Roberts		Stašinskis	Riga East Clinical University Hospital, Riga
Jonas		Surkus	Lithuanian University of Health Sciences, Kaunas, Lithuania
Irma		van den Hengel-Koot	Dutch Poisons Information Center, University Medical Center, Utrecht University, Utrecht, The Netherlands
Federico		Vigorita	San Gerardo Hospital [U.O.S. Pronto Soccorso], ASST- Monza, Italy
Severin	B	Vogt	Clinical Pharmacology and Toxicology, University Hospital and University of Basel, Basel, Switzerland
William	Stephen	Waring	Acute Medical Unit, York Teaching Hospitals NHS Foundation Trust

			York, UK
Ioanna		Yiasemi	Monitoring Department, Cyprus National Addictions Authority, Nicosia, Cyprus
Sergej		Zacharov	Toxicological Information Centre, Department of Occupational Medicine, 1st Faculty of Medicine, Charles University and General University Hospital, Na Bojisti 1, 120 00 Prague 2, Czech Republic
Tobias		Zellner	Department of Clinical Toxicology, Klinikum rechts der Isar, Technical University of Munich, Germany
